# Physical frailty and functional status in patients with advanced kidney disease: a protocol for a systematic review

**DOI:** 10.1186/s13643-017-0536-1

**Published:** 2017-07-06

**Authors:** Kendra E. Brett, Alexandria Bennett, Lindsay J. Ritchie, Greg A. Knoll

**Affiliations:** 1 Clinical Epidemiology Program, Ottawa Hospital Research Institute, 501 Smyth Road, Ottawa, Ontario K1H 8L6 Canada; 2 Department of Medicine, University of Ottawa, Ottawa, Ontario Canada; 3 Division of Nephrology, Kidney Research Centre, Ottawa Hospital Research Institute, Ottawa, Canada

**Keywords:** Frailty, Functional status, Kidney transplantation, Chronic kidney disease, Systematic review

## Abstract

**Background:**

Predicting outcomes in those with chronic kidney disease or following transplantation is challenging, and current models lack detailed patient-level information. Frailty and poor functional status are risk factors for adverse patient outcomes that may be useful additions to prognostic tools in patients with chronic kidney disease. The purpose of this systematic review is to examine whether frailty or functional status are associated with increased risk of mortality or adverse clinical outcomes in patients with advanced kidney disease.

**Methods/design:**

We will conduct a systematic review to identify and evaluate studies linking frailty and functional status with patient outcomes in populations with advanced kidney disease. We will search MEDLINE, Embase, and the Cochrane Central Register for Controlled Trials. Two reviewers will conduct all screening and data extraction independently. A modified version of the Quality In Prognosis Studies tool will be used to evaluate the quality of the studies. If meta-analysis of outcome data is possible, a random effects model will be used.

**Discussion:**

The results of this review will inform the development, selection, and validation of appropriate metrics needed to improve prognostication in patients with chronic kidney disease.

**Systematic review registration:**

PROSPERO CRD42016045251

**Electronic supplementary material:**

The online version of this article (doi:10.1186/s13643-017-0536-1) contains supplementary material, which is available to authorized users.

## Background

Approximately three million Canadians have chronic kidney disease (CKD) [1]. Once this condition progresses to end-stage renal disease (ESRD), either dialysis or kidney transplantation is required to sustain life. Kidney transplantation is the preferred treatment since it improves quality of life, prolongs survival, and is less costly compared to dialysis [2–4]. Although there are few absolute contraindications, patient selection for transplantation can be challenging especially in older individuals with multiple comorbid conditions. Models have been developed to estimate survival post-transplant and assist clinicians with patient selection, but their predictive ability at the patient-level has been modest [5–8]. Most of these prediction models have been developed using large administrative datasets and lack granular patient details such as the ability to perform activities of daily living and functional or cognitive status. There is a need to develop novel predictive tools that will better inform both patients and clinicians regarding appropriate candidate selection for kidney transplantation.

Frailty and functional status are emerging as risk factors for adverse outcomes in patients with ESRD [5–7, 9–11]. The physical frailty phenotype is a multidimensional syndrome that has been defined as “*a medical syndrome with multiple causes and contributors that is characterized by diminished strength, endurance, and reduced physiological function that increases an individual’s vulnerability for developing increased dependency and/or death*” [12]. Functional status is defined as an individual’s ability to carry out the normal activities of daily living required to meet basic needs, fulfill usual roles, and maintain health and well-being [13]. Patients with CKD have a higher risk of being frail when compared to those without CKD [14–16], and the initiation of dialysis has been linked to substantial decline in functional status in elderly patients with ESRD [17]. Incorporating frailty or functional status, in addition to conventional risk factors, into the evaluation of CKD patients for transplantation may improve our ability to predict post-transplant survival and enhance the selection of appropriate transplant candidates. The purpose of this study is to systematically identify and review all relevant studies that explore the link between frailty or functional status and adverse clinical outcomes in patients with CKD.

## Research objectives

The aim of this study is to give an overview of all currently available evidence regarding the relation of frailty and functional status with mortality or adverse clinical events in a population with CKD. Specific objectives are the following: first, we will explore whether frailty or functional status are associated with increased risk of mortality or adverse clinical outcomes in patients with advanced kidney disease. Secondly, we will determine which instruments or tools for measuring frailty or functional status have undergone validity or reliability testing in this population.

## Methods

### Study eligibility criteria

Peer- reviewed, published articles will be selected according to the criteria outlined below.

#### Study designs

We will include retrospective and prospective studies (cohort, case-control, cross-sectional, and longitudinal studies) and case series with a minimum of 20 cases. Interventional studies (randomized and non-randomized) will only be included if the intervention could not have influenced the outcomes of interest. Commentaries, reviews, letters, and editorials will be excluded.

#### Participants

We will include studies examining adult (18 or older) male or female patients with kidney disease, including but not limited to CKD, ESRD, patients on dialysis, and those on the waiting list for or who have received a kidney transplant. We will include studies addressing both adults and children if data provided for adults are reported separately.

We will exclude patients with acute kidney injury/failure and studies where the primary focus is not patients with kidney disease. We will also exclude articles involving CKD patients who also have another disease or condition for which they were specifically recruited or studied. Articles will be included if patient comorbidities are listed in addition to the kidney disease, but articles that focus on a specific additional disease (e.g., CKD with acute stroke) will be excluded.

#### Assessments: frailty and functional status

Of interest are the studies that measure patient frailty and/or functional status. Assessments of frailty will be considered if a specific frailty measure/tool is used, such as the Fried criteria [18], or if one or more of the individual domains of physical frailty are measured, such as sarcopenia, slowness, weakness, poor endurance/exhaustion, or low physical activity. Assessments of functional status will be considered if a specific assessment tool for functional status is used, such as the SF-36 physical functioning scale, or if the physical measures of functional performance are considered, such as activities of daily living (ADL), or intermediate activities to enable those needs (IADL) [13]. The assessment tools can be objective or subjective measures and directly measured or self-reported assessments. Articles will be excluded if patient frailty or functional status is only measured post-transplant or if the assessment tools are specifically focused on the following: the cognitive side of frailty, nutritional status, mood or mental health symptoms only, or social relations or support. If an assessment tool is comprised of multiple components and one of the excluded topics is included as one of the components, then the tool will still be included if physical frailty or functional status are also a component of the tool.

Given the variety of potential measures of frailty and functional status, several different types of comparisons will be considered. We recognize that there is the possibility that frailty and functional status may be assessed along a continuous scale, that patients may be grouped into two or more categories (e.g., frail vs. not frail, or low, intermediate or high physical functioning), or these measurements may be presented as a progression over time.

#### Outcomes

We are interested in reported clinical outcomes that are related to successful/favorable outcomes in patients with kidney disease or following a kidney transplant. The primary outcome of interest is mortality. As mortality can be defined at a number of time points, all time points for mortality will be acceptable and will be categorized and described in the review. The following (secondary) outcomes will also be considered:

- Adverse clinical outcomes including:
  - ○Kidney transplant failure or rejection
  - ○Delayed graft function post-transplant
  - ○Hospitalization (including length of stay)
  - ○Infection
  - ○Risk of cardiovascular disease/events (i.e., stroke, myocardial infarction)
  - ○Transplant candidacy/wait list deactivation
  - ○Length of time on the wait list
  - ○Treatment or medication non-adherence
- Measures of kidney function, such as glomerular filtration rate
- Quality of life

Outcome data will not be restricted to a specific format of data (e.g., dichotomous, continuous), and as such, we will extract the data as it is reported in the included studies.

#### Validity and reliability assessments

We will also include studies that may not have reported clinical outcomes but have measured the validity or reliability of the frailty or functional status tools in a population with CKD. Reliability testing, defined as the degree to which an instrument is free from measurement error, will be considered if either internal consistency reliability or test-retest reliability is reported [18]. Validity, defined as the degree to which an instrument measures the concept it is intended to measure, will be considered if content validity, construct validity (congruent or known group), or responsiveness are reported [18].

#### Timing

There will be no restrictions for the length of follow-up for the studies.

#### Setting

There will be no restrictions by the type of setting.

### Search strategy

A comprehensive electronic literature search will be conducted in MEDLINE, Embase, and the Cochrane Central Register for Controlled Trials (CENTRAL) from inception to October 2016. The reference lists of included studies and relevant reviews will be scanned for additional studies that were not captured by the search. The search strategy for MEDLINE will be developed with the assistance of a medical librarian experienced in systematic reviews. After the MEDLINE strategy is finalized, it will be adapted to the syntax and subject headings of the other databases. A structured search strategy will be based on controlled vocabulary and relevant key terms (Additional file 1). Key search terms will include end-stage renal disease, frailty, sarcopenia, functional status, and activities of daily living. Language of publication will be restricted to the English language. This review has been registered with PROSPERO (CRD42016045251).

### Study screening and inclusion

Literature search results will be uploaded to EndNote X7 software, which will be used to find and remove duplicates. References will be sorted alphabetically by author and exported to Microsoft Word for screening of the titles and abstracts. Additional duplicates that may have been missed by the referencing software will be identified and removed during screening. The screening process will take place in two stages: screening of titles and abstracts (stage 1) and screening the full text of the articles (stage 2). For both stages, two independent reviewers will screen each reference against the eligibility criteria outlined in the previous section. For all titles and abstracts that appear potentially eligible, we will obtain full-text reports. If no abstract is available for a given citation, then the full text will be obtained unless the article can be confidently excluded by its title alone. Prior to the formal screening process, the reviewers will participate in a short pilot exercise to identify and address any inconsistencies in the application of the screening criteria. A third reviewer will reconcile any disagreements between the two reviewers regarding an article’s inclusion status. We will record the reasons for excluding studies during stage 2 of the screening process. The study selection process will be summarized using a Preferred Reporting Items for Systematic Reviews and Meta-Analyses (PRISMA) flow diagram [19].

### Data extraction

Each eligible study will undergo data extraction by two independent reviewers using a pre-designed form in Microsoft Excel and a detailed instruction manual. To ensure consistency across reviewers and that the form captures all relevant information, the form will be pilot-tested on a small number of studies before starting the data extraction process. Any discrepancies in data extraction between the two reviewers that cannot be resolved by discussion will be adjudicated by a third reviewer.

If a study is reported in more than one publication, we will take one of the two approaches, depending on the nature of the reports. If a full journal article plus one or more conference abstracts are available, we will extract the data from all reports directly into a single data extraction form, as it is likely that the majority of the information will be obtained for the journal article. If there are two or more detailed journal articles, then the data will be extracted from each report separately, and then, the information will be combined.

We will extract information pertaining to the following: study identification (author, year of publication, number and location of centers, funding, and journal), study design (type of study, methods, sample size, eligibility criteria, and duration of follow-up), patient population [age, gender, ethnicity, duration and type of kidney disease, length of time since transplant (if applicable), type of donor (if applicable), comorbidities, duration of renal replacement therapy], details about the comparator group(s) (how were the groups compared), details about the assessment of frailty or functional status [what was assessed (name of the measurement tool or specific domain that was measured), timing of the measurement (when was it measured, length of time between measurement and transplant or outcomes of interest, frequency of the assessment), and the details of the assessment tool (objective or subjective, whether it was a categorical or continuous assessment, the administrative time, scoring/categorization)], the outcomes of interest (definitions, measurement methods, time since transplant/follow-up interval, data, adjusted and unadjusted point estimates), and the details of the reliability and validity testing (details on how testing was conducted and statistical details).

### Risk of bias

For studies reporting an outcome of interest, the methodological quality will be evaluated using a modified version of the Quality In Prognosis Studies (QUIPS) tool [20–22]. Six domains will be evaluated within each article: study participation, study attrition, frailty or functional status instrument measurement, outcome measurement, study confounding, and statistical analysis and reporting. For each domain, a number of prompting questions will enable the assessment of the domain as having a high, moderate, or low risk of bias. The risk of bias for each included study will be assessed by one member of the research team and verified by a second member. Disagreements will be resolved by consensus or by a third member, if needed.

### Evidence synthesis and data analysis

Information summarizing the characteristics and findings of all included studies will be presented in the text and in tables. The information pertaining to the frailty and functional status measures will be presented and discussed separately. In addition, the studies that include validity or reliability testing will be presented separately from those linking frailty or functional status to a clinical outcome. A summary table will provide the relevant details for all included studies, including study identification, the details of the frailty or functional status measurement, and the outcomes or reliability/validity details. The studies will be grouped depending on whether they are measuring frailty, functional status, or both, or whether they test the reliability/validity of the assessment tools. Additional tables will be used to summarize the details of all of the frailty and functional status measurement tools used in the included studies, which will include the following: a brief description of how the tool measures frailty or functional status, the patient populations in which the tool was used (sample size, age, type of kidney disease), and whether the validity or reliability were tested in the kidney disease population.

We anticipate a large degree of heterogeneity between the studies in terms of assessment tools, populations, outcomes, and study design, and we do not believe that the statistical combination of the studies will be a prominent component of this review. As such, a narrative synthesis will be used to summarize the predictive value of the frailty and functional status measures. In this summary, the available evidence will be discussed by taking into account the number of studies evaluating this factor, the methodological quality of these studies, and the consistency of the available results. We will retain studies of any level of risk of bias in the narrative synthesis.

It is unlikely that there will be sufficiently similar studies to conduct a meta-analysis; however, this will be considered once the data is collected. Summary measures for the main outcome (mortality) may be in the form of relative risk or hazard ratio (adjusted or unadjusted). For the other outcomes, the summary measures may be reported in a variety of ways, including mean differences, relative risk, or hazard ratios. Where possible, appropriate meta-analytic methods will be employed to combine data from similar studies. Studies will be grouped by the stage of kidney disease at the time of the frailty or functional status measurement, in order to account for differences in disease severity. If meta-analysis is possible, the effect of frailty or functional status will be stratified by outcome, using a random effects model. Where studies have reported time-to-event analyses, meta-analysis using the extracted hazard ratios and their variances will be undertaken. Evidence from studies with different designs will not be quantitatively combined but presented separately. Visual representation of results in Forest plots without pooling may also be considered.

We will report on the presence of reliability and validity testing, rather than conducting a quality assessment of these measures. A table will be prepared to show which metrics underwent validity or reliability testing, and descriptive statistics will be compiled, including a summary of the available results for the reliability or validity tests for each measure.

## Discussion

The aim of this systematic review is to examine the link between frailty and functional status measures and clinical outcomes in patients with advanced kidney disease. We hope to identify tools that can help predict which patients have an increased vulnerability to future decline, which will in turn help clinicians to identify appropriate renal transplant candidates based on patient-specific factors. Reliable and valid predictive tools are necessary to determine who will benefit from and who should be referred for kidney transplantation. Current prognostic tools are not sensitive or specific enough to predict how long a patient will survive, and validated prognostic tools that incorporate important patient information, such as frailty or functional status, may be useful for influencing treatment decisions.

## Reporting of the review

We used the PRISMA for protocols (PRISMA-P) checklist for reporting this protocol; this is included as an additional file (Additional file 2). The results of this review will be reported using the PRISMA statement [19]. This protocol does not update any previously conducted systematic review. Any changes made to this protocol when conducting the review will be outlined in the review’s manuscript.

## Additional files

Additional file 1: Search strategy. The data provided shows the comprehensive search strategy for the MEDLINE database. (DOCX 14 kb)

Additional file 2: PRISMA-P checklist. The data provided shows a completed copy of the PRISMA-P checklist to guide readers in assessing the quality of the current protocol. (DOCX 38 kb)

## Abbreviations

ADL: Activities of daily living
CENTRAL: Cochrane Central Register for Controlled Trials
CKD: Chronic kidney disease
ESRD: End-stage renal disease
IADL: Intermediate activities of daily living
PRISMA: Preferred Reporting Items for Systematic Reviews and Meta-Analyses
PRISMA-P: Preferred Reporting Items for Systematic Review and Meta-Analysis Protocols
QUIPS: Quality In Prognosis Studies
