# Supplementary material for: Physical frailty and functional status in patients with advanced kidney disease: a protocol for a systematic review
Source: Syst Rev. 2017 Jul 6;6:133. doi: 10.1186/s13643-017-0536-1 (PMC5501003; doi:10.1186/s13643-017-0536-1)
Supplement: Supplementary file 1 — Search strategy. The data provided shows the comprehensive search strategy for the MEDLINE database. (DOCX 14 kb) [file 13643_2017_536_MOESM1_ESM.docx]

Search Strategy for MEDLINE

1. renal insufficiency/ or renal insufficiency, chronic/ or kidney failure, chronic/

2. renal replacement therapy/ or renal dialysis/ or peritoneal dialysis/ or kidney transplantation/

3. ((renal or kidney*) adj2 (transplant* or graft* or replac* or artificial* or allograft* or dialys*)).tw,kw.

4. h?emodialysis.tw,kw.

5. esrd.tw,kw.

6. esrf.tw,kw.

7. pre-esrd.tw,kw.

8. CKD.tw,kw.

9. ((kidney* or renal) adj (failure* or disease* or insufficien* or disorder*)).tw,kw.

10. ((kidney* or renal) adj replacement therap*).tw,kw.

11. 1 or 2 or 3 or 4 or 5 or 6 or 7 or 8 or 9 or 10

12. Frail Elderly/

13. Geriatric Assessment/

14. muscle weakness/

15. sarcopenia/

16. Fatigue/

17. frail* or strength.tw,kw.

18. Sarcopenia*.tw,kw.

19. Fatigue.ti,kw.

20. (muscle* adj2 weak*).tw.

21. Walking/

22. "Activities of Daily Living"/

23. mobility limitation/

24. (functional adj (status or performance or capacity or dependence or independence or ability or decline or disability)).tw,kw.

25. (physical adj (performance or capacity or ability or disability or function* or fatigue)).tw,kw.

26. ((limit* or decline) adj2 mobility).tw,kw.

27. activit* of daily living.tw,kw.

28. daily living activit*.tw,kw.

29. 12 or 13 or 14 or 15 or 16 or 17 or 18 or 19 or 20 or 21 or 22 or 23 or 24 or 25 or 26 or 27 or 28

30. 11 and 29

31. "reproducibility of results"/

32. "Predictive Value of Tests"/

33. Psychometrics/

34. (reliability or reproducib* or measurement* or measure or scale* or index or indices or predict* or psychometric* or instrument* or score* or validity or validat* or test or prognos*).tw,kw.

35. Validation Studies/

36. phenotype*.tw.

37. risk.tw.

38. 31 or 32 or 33 or 34 or 35 or 36 or 37

39. 30 and 38

40. limit 39 to english
